# Occupational Health of Education Personnel—The Role of Job Crafting and Other Control Strategies on Healthy Ageing at Work

**DOI:** 10.3390/ijerph192315970

**Published:** 2022-11-30

**Authors:** Min-Chien Tsai, Sy-Feng Wang, Nicola J. Gray, Didier Jourdan

**Affiliations:** 1 Department of Psychology, Fu Jen Catholic University, New Taipei City 242062, Taiwan; 2 School of Applied Sciences, University of Huddersfield, Huddersfield HD1 3DH, UK; 3 ACTé EA 4281 Research Group, Université Clermont Auvergne, F-63000 Clermont-Ferrand, France

**Keywords:** job crafting, primary and secondary control, healthy ageing, well-being, teachers

## Abstract

This article looks at the strategies that influence healthy ageing at work from the motivational theory of life span development (MTD). It aims to better understand the influence of job crafting as a selective primary control, help-seeking as a compensatory primary control, positive reappraisal as a selective secondary control, and downward social comparison and downgrading expectation as a compensatory secondary control on healthy ageing at work (work engagement, health, and motivation to continue working after retirement). A total of 386 educational personnel participated in the study. This study used hierarchical regression analysis to test incremental validity, supplemented with confirmatory factor analysis and structural equation modelling as a solution to solve the potential error problems caused. The results show that job crafting is positively correlated with healthy ageing at work. Positive reappraisal and downward social comparison showed incremental validity in predicting healthy ageing at work beyond job crafting among the middle-aged group (45–65-years-old); in particular, positive reappraisal was the determinant of healthy ageing at work among the middle-aged group. However, both help-seeking and downgrading expectation did not show incremental validity. This study can contribute to the evolution of career development interventions and human resource management focused on supporting older people at work.

## 1. Introduction

As they age, people become wiser and more experienced, on the one hand; on the other hand, however, age brings physical and psychological changes, limitations and challenges. Some scholars have developed the concept of successful ageing at work or healthy ageing at work. This extends existing notions of successful ageing to the paid workplace context, i.e., maintaining a high level of health, occupational well-being, motivation, and work capacity at work, where work capacity refers to whether or not one continues to work [1,2].

People ranging from about 45–65-years-old (middle-aged) are at a maintained stage, and are ready to move forward to the final stage of their career—the disengagement stage (retirement) [3]. The study of the psychological and social processes involved in the final stages of the working career is an essential field of research [4].

Throughout their careers, workers develop strategies for adapting to the workplace, but even more so in the latter part of their working lives. The Motivational Theory of Life Span Development (MTD) proposes that the individual’s attempts to regulate his or her own development is organised in cycles of action around the pursuit of developmental goals. People adopt control strategies in order to play an active role in choosing, achieving, or disengaging from their goals in coping with various life span developmental tasks [5]. Job design, or job redesign, refers to the arrangement of tasks, activities, relationships, and responsibilities by an organisation [6]. It is not only the organisation that does something, but also the workers themselves. Therefore, in contrast to organisational job design or job redesign, which is top-down, job crafting is a bottom-up process of change initiated by workers [7]. If, at first, job crafting research was not tailored to middle-aged people [8,9], there is now a marked interest in it [10,11]. This worker-initiated adaptation process could be considered as a control strategy. Job crafting, help-seeking, positive reappraisal, downward social comparison, and downgrading expectation are different control strategies. The objectives of this study are to better understand the influence of these control strategies on healthy ageing at work. Following Zacher [12], healthy ageing at work is described here through three dimensions: work engagement (occupational well-being), general health (occupational health), and motivation to continue working (work motivation).

In this work, we focused on education staff. The latter part of the career of education professionals can be a major challenge to their well-being [13,14], although intellectually stimulating work is associated with higher levels of successful ageing [15]. Recently, the COVID-19 crisis showed that older teachers were more likely to report high levels of anxiety and stress compared to younger professionals [16,17,18]. What is at stake here, therefore, is not only the health of education personnel themselves, but also that of their students. Better well-being and lower depression levels in teachers are linked to better student well-being [19,20]. Moreover, a negative relationship between teachers’ emotional exhaustion and students’ academic development has also been reported [21,22].

The first report of the International Barometer of the Health and Well-Being of Education Personnel, published in November 2021 [23], showed a very high rate of dissatisfaction concerning career development opportunities among education professionals. In the conclusion, the authors stated: “The COVID crisis exacerbated pre-existing problems in the education sector: effort/reward and work/life imbalance, lack of training, of career development and of hierarchical support, and school violence”. The post-COVID-19 context makes it all the more important to identify the conditions that enable education personnel to age well at work. The importance of the life span development perspective was also highlighted.

### 1.1. Theoretical Background

As the main purpose of this study is to investigate the determinants of ageing at work among middle-aged education professionals, we use a dynamic theoretical framework. We have chosen the motivational theory of life span development (MTD), which has previously been used in the context of healthy ageing [24,25]. Heckhausen and Schulz [26] integrated the primary/secondary control perspective of Rothbaum et al. [27], and selection/compensation from the model of selective optimisation with compensation [28], into a two-dimensional model (2 × 2) with four control strategies: (1) selective primary control, (2) compensatory primary control, (3) selective secondary control, and (4) compensatory secondary control [24] (Table 1). *Selective primary control* refers to the investment of internal resources for a chosen goal, such as investing time and effort in learning new knowledge and skills. In this paper, we focus on a well-defined control strategy known as job crafting [29]. *Compensatory primary control* refers to the use of external resources, such as using special technical aids and seeking help from colleagues. *Selective secondary control* refers to the internal representations for a chosen goal, such as retaining a goal commitment in order to avoid distraction from other attractive goals. *Compensatory secondary control* refers to the buffering of negative effects of failure or losses on motivational resources, such as downward social comparison, disengagement from the previous goal, or changing to another goal [6,24,30]. The MTD treats control strategies as the role of motivation regulation and encompasses behaviours, actions, or thoughts to influence their choice, effort, or persistence [31].

Primary control remains stable at a high level throughout the life span, and primary control capacity reaches the highest level in midlife and then begins to show a decline in old age due to age-related limitations. Secondary control reaches the highest level in old age [6,32]. Primary control capacity for younger professionals was still highly adequate, so we focused on middle-aged professionals for this reason. Moreover, a process perspective was provided from the MTD. According to the proposed framework from this theory, the selective mechanism was activated first if someone encountered failure, and the compensatory mechanism promoted its functions on maintenance, enhancement, and recovery [24].

**Table 1:** Potential strategies that influence healthy ageing at work.

Control Strategy	Definition of the Control Strategy	Potential Strategy	Definition	Influence on Healthy Ageing at Work
Selective Primary Control	Selective primary control refers to the investment of internal resources for a chosen goal [24].	Job Crafting (Developmental Crafting and Utilisation Crafting)	Developmental crafting refers to learning new skills or growth; Utilisation crafting refers to utilising existing skills and knowledge [25].	Work engagement (Occupational well-being) [33,34,35,36,37]; Health [38]; Motivation to work until maximum retirement age [11].
Compensatory Primary Control	Compensatory primary control refers to the use of external resources [24].	Help-Seeking	Find someone else to help with the task [39].	Work engagement [1].
Selective Secondary Control	Selective secondary control refers to the internal representations for a chosen goal [24].	Positive Reappraisal	Positive reappraisal reflects positive meaning in the face of adversity [40].	Well-being [41,42,43,44]; Health [43,45].
Compensatory Secondary Control	Compensatory secondary control refers to the buffering negative effects of failure or losses on motivational resources [24].	Downward Social Comparison	Downward social comparison with a standard worse off than the comparer [46].	Well-being [42,47]; Health [48].
		Downgrading Expectation	Expect less of yourself [39].	Health [43].

### 1.2. Hypothesis Development

Our study aims to identify the strategies that could influence healthy ageing at work. Our general hypothesis is that job crafting and control strategies influence healthy ageing at work. More precisely, this study is based on the assumption that (1) primary control (especially job crafting) may precede secondary control and that (2) the selection mechanism may precede the compensation mechanism, according to a process perspective of the MTD [5,30,32].

#### 1.2.1. Job Crafting as a Selective Primary Control Strategy

A concept applicable to selective primary control is ‘job crafting’, proposed by Wrzesniewski and Dutton [48], where job crafting is defined as self-initiated changes in job characteristics, job relationships, and job meaning [7]. Kooij et al. [29] proposed three dimensions of middle-aged job crafting: *Accommodative crafting* refers to regulating losses, such as hiring an assistant; *Developmental crafting* refers to learning new skills or growth, such as attending training to sharpen skills and taking tough challenges; and *Utilisation crafting* refers to utilising existing skills and knowledge, such as focusing on personal strengths and interests. Selective primary control includes investing effort and time, developing relevant skills and abilities, and fighting difficulties [49]. Developmental crafting and utilisation crafting are similar to selective primary control; this study considered job crafting as a selective primary control strategy for this reason.

Job crafting has been shown to be a significant predictor of work engagement [33,34,35,36,37]. Moreover, promotion-focused job crafting was shown to be positive for health, and prevention-focused job crafting was negative for health [38]. It has been found that exhibiting more job crafting behaviours was associated with more middle-aged professionals being willing to work until their maximum retirement age [11].

**Hypothesis** **1** **(H1).**  *Job crafting is positively related with (a) work engagement, (b) general health, and (c) motivation to continue working after retirement among middle-aged education professionals.*

#### 1.2.2. Help-Seeking as a Compensatory Primary Control Strategy

Compensation control is a mechanism of using external resources to maintain and remedy after loss, failure, and frustration, e.g., seeking advice and help [24], which is particularly beneficial in middle age, and for healthy ageing at work, to respond to age-related changes. Help-seeking is considered a kind of compensatory primary control strategy [30]. Zacher et al. [1] argued that compensation strategies had positive effects on work engagement. Liu et al. [50] found that job stressors increased help-seeking. For middle-aged professionals experiencing more and more age-related challenges, help-seeking behaviours should increase. There has been little research conducted on the effect of help-seeking on each dimension of healthy ageing at work, so this remains unclear. Given the importance of compensatory primary controls for middle-aged professionals, it is worth exploring its impact.

According to the MTD, selective primary control for goal striving was used prior to compensatory primary control and selective secondary control [25], and the following hypothesis is based on this argument to test the incremental validity of other control strategies beyond job crafting (selective primary control).

**Hypothesis** **2** **(H2).**  *Help-seeking has incremental validity for (a) work engagement, (b) general health, and (c) motivation to continue working after retirement beyond job crafting among middle-aged education professionals.*

#### 1.2.3. Positive Reappraisal as a Selective Secondary Control Strategy

It was found that, when people were frustrated, they were more likely to use a secondary control strategy [27]. The functions of secondary control are to enhance primary control along the life span [51]. Positive reappraisal is considered a kind of compensatory primary control strategy [44], and it refers to finding positive meaning in the face of adversity [40].

Nowlan et al. [52] found that positive reappraisal was used more frequently by older adults than younger adults, and that positive reappraisal showed a significant positive correlation with well-being. Similar results indicate that positive reappraisal as a compensatory primary control had a positive relationship with well-being among older adults [41,42,43,44]. Low et al. [45] reported that staying positive could keep people psychologically and physically healthy. Hall et al. [43] also found that positive reappraisal predicted a higher level of health status.

**Hypothesis** **3** **(H3).**  *Positive reappraisal has incremental validity for (a) work engagement, (b) general health, and (c) motivation to continue working after retirement beyond job crafting among middle-aged education professionals.*

#### 1.2.4. Downward Social Comparison and Downgrading Expectation as Compensatory Secondary Control Strategies

Based on the MTD, compensatory secondary control is incremental throughout the life span [5,30]. On the other hand, compensatory secondary control strategies have been used for goal disengagement and to help people to protect themselves, as well as distancing from a goal [25]. For this reason, this study did not propose that compensatory secondary control has incremental validity for motivation to continue working after retirement.

Downward social comparison and downgrading expectation are both compensatory secondary control strategies [5]. Downward social comparison happens with a standard who is considered ‘worse off’ than the person making the comparison [46]. Downward social comparison was related to well-being at low levels of perceived control [47]. Du et al. [42] also found that social comparison had a positive relationship with their well-being among older adults. Sayag and Kavé [48] found that older adults who selected their peers as a comparison group reported better health status than those who selected younger adults. Hall et al. [43] found that downgrading goal importance predicted a higher level of health status. Wrosch et al. [44] found that downgrading expectation was negatively related to well-being, so this study did not propose that downgrading expectation has incremental validity for work engagement.

**Hypothesis** **4** **(H4).**  *Downward social comparison has incremental validity for (a) work engagement and (b) general health beyond job crafting among middle-aged; downgrading expectation has incremental validity for (c) general health beyond job crafting among middle-aged education professionals.*

To the best of our knowledge, prior studies on the motivational theory of life span development (MTD) have three weaknesses and limitations. First, the importance of neither primary control nor secondary control was clearly supported, and lacked consistent comparative evidence of different age groups, as identified by Wong et al. [53]; for example, the teachers in young adulthood used selective secondary control strategies more frequently than their younger and older colleagues. Second, there is little research that examines the control strategies of educational staff in the latter part of their career [54]. Third, the scale of the MTD was development over 20 years ago [55], and there are a number of concepts that were developed later that can be integrated, such as job crafting. In this article, to contribute to the knowledge in three important ways, first, we describe the results surrounding the role of control strategies based on the MTD for the middle-aged and younger groups on healthy ageing at work, with additional evidence on the theory expected. Second, our outcome variables were for healthy ageing at work, to echo the context of healthy ageing of the MTD and practical needs. Third, we integrated the job crafting and selective primary control strategy to provide a new viewpoint of the MTD.

## 2. Materials and Methods

### 2.1. Participants and Procedure

Data were collected between January and March 2022. In order to be able to make comparisons, both middle-aged and younger education professionals were included. We used the standards of the Ministry of Labor for the definition of ‘middle-aged’, i.e., 45 to 65-years-old [56], to inform our inclusion criteria. We included participants who reported working in elementary, middle, and high schools as principals, teachers, and staff. The schools were involved in the Health Promoting Schools scheme and have strengthened their conditions to be healthy living, learning, and workplace settings. They were contacted, then snowball sampling was used to reach 30 schools. Upon the approval of the 30 school principals, 617 teachers and staff were invited to complete an online survey. A question posing a trap was inserted in the questionnaire: “This question is a test of the quality of the questionnaire, please do not answer this question”. This was used as indicator of data quality [57], showing whether the participants noted that they were asked to ignore one question.

### 2.2. Measures

This self-report study was based on an online survey. The language used was Chinese. The predictor and outcome variables are summarised below and in Table 2.

#### 2.2.1. Predictor Variables

The study used and revised previously developed instructions to assess task-specific control strategies [39]. Participants were asked to identify a task/activity that they found difficult, due to age, and then to report the frequency of specific control strategies they used to deal with the task/activity they found difficult.

Job crafting was assessed using an existing ten-item scale from Kooij et al. [58]. Help-Seeking was assessed with a three-item scale from Haynes et al. [39]. Positive Reappraisal was also assessed with a three-item scale from Haynes et al. [39]. Downward Social Comparison was assessed with a four-item scale from Haynes et al. [39]. Downgrading Expectation was assessed with a three-item scale from Haynes et al. [39].

#### 2.2.2. Outcome Variables

Work Engagement was assessed with a three-item scale from Schaufeli et al. [59]. General Health was assessed with a five-item scale from Ware and Sherbourne [60]. Motivation to Continue Working after Retirement was assessed using a modified version of a three-item scale from Lichtenthaler and Fischbach [61].

#### 2.2.3. Control Variable

Gender has been found to be linked to the work engagement of teachers [62], the health status of teachers [63], and whether to retire or extend work lives [64,65]. Since gender is associated with all dimensions of healthy ageing at work, it was included as a control variable.

#### 2.2.4. Items for Psychological Separation

To avoid people immediately answering questions related to outcome variables after questions about predictor variables, irrelevant items were introduced to minimise common method bias [66]. A total of six questions were selected from Tseng et al. [67] on intuitive responses to emotion words (in Chinese), which participants were asked to fill in.

### 2.3. Data Analysis

We used SPSS version 20 and Mplus version 8. Schafer [68] stated that if missing data are small, and less than 5%, single imputation may tend to the accurate value. Mean imputation was used for one missing datum of general health and for five missing data of age. Mode imputation was used for three missing data of gender and three missing data of professional identity.

The measure of job crafting towards utilisation and development with a ten-item, three-parcel approach with factorial allocation was used [69]. Step 1 computed one-factor loadings with 10 items; Step 2, ordered according to the factor loadings, in order from the highest to the lowest; Step 3, allocated the first parcel with the 1st, 6th, and 7th ordered items; the second parcel with the 2nd, 5th, and 8th ordered items; and the third parcel with the 3rd, 4th, 9th, and 10th ordered items.

A multiple regression approach was used to test our hypothesis for incremental validity. Wang and Eastwick [70] indicated that this common approach has a potential problem with type I error; therefore, confirmatory factor analysis (CFA) and structural equation modelling (SEM) analysis were needed to solve the problem. According to their suggestions, the incremental validity testing analysis in this study was designed as follows. First, we used the CFA to provide evidence for separate constructs of each focal predictor (other control strategies) and the covariate variable (job crafting) by Mplus. Next, we tested a series of hierarchical regression. Finally, we conducted SEM analysis with latent variables to test the complete model instead of part of the model by Mplus.

## 3. Results

A total of 442 participants answered the survey, which corresponds to a 71.6% response rate. Overall, 49 respondents who answered the trap question and seven respondents who did not answer any of the items in the outcome variables were excluded. The final sample therefore included 386 participants, with more females (67.1%) than males (32.9%), and roughly equal proportions of middle-aged (49.5%) and younger respondents (50.5%). The age of the participants ranged from 22 to 65 years; on average, they were 44-years-old (*SD* = 8.72). The majority of the participants worked in elementary schools (73.6%); others worked in a middle school (17.6%) or high school (8.8%). Most of the participants were formal teachers (56.7%), while the remainder were principals (4.1%), acting teachers (14%), part-time teachers (1%), administrative directors (9.6%), non-specified staff (10.9%), and other (3.6%). Almost three-quarters (74.1%) of the participants had not yet met the retirement conditions, while the rest had done so.

### 3.1. Preliminary Analyses

Table 3 presents the descriptive statistics and intercorrelations of the study variables. As expected, job crafting as a selective primary control was positively related to work engagement (*r* = 0.39, *p* < 0.001), general health (*r* = 0.26, *p* < 0.001), and motivation to continue working (r = 0.21, *p* < 0.001). Positive reappraisal as a selective secondary control strategy was positively related to work engagement (*r* = 0.41, *p* < 0.001), general health (*r* = 0.37, *p* < 0.001), and motivation to continue working (*r* = 0.21, *p* < 0.001). Downward social comparison as a compensatory secondary control strategy was positively related to work engagement (*r* = 0.27, *p* < 0.001), general health (*r* = 0.26, *p* < 0.001), and motivation to continue working (*r* = 0.17, *p* < 0.01). However, help-seeking as a compensatory primary control strategy was only positively related to general health (*r* = 0.11, *p* < 0.05). Moreover, downgrading expectation as another compensatory secondary control strategy was not positively related to any outcome variables. Gender was positively related to downward social comparison (*r* = 0.10, *p* < 0.05).

In addition, we also examined the Pearson correlation analysis between the focal variables for the middle-aged group and younger group, respectively. The results demonstrate that job crafting was positively associated with work engagement (middle-aged group for *r* = 0.36, *p* < 0.001; younger group for *r* = 0.43, *p* < 0.001), general health (middle-aged group for *r* = 0.18, *p* < 0.05; younger group for *r* = 0.35, *p* < 0.001), and motivation to continue working (middle-aged group for *r* = 0.26, *p* < 0.001; younger group for *r* = 0.17, *p* < 0.05) for both groups. Positive reappraisal was positively associated with work engagement (middle-aged group for *r* = 0.46, *p* < 0.001; younger group for *r* = 0.40, *p* < 0.001), general health (middle-aged group for *r* = 0.41, *p* < 0.001; younger group for *r* = 0.34, *p* < 0.001), and motivation to continue working (middle-aged group for *r* = 0.29, *p* < 0.001; younger group for *r* = 0.14, *p* < 0.05) for both groups. Downward social comparison was positively related to work engagement (middle-aged group for *r* = 0.27, *p* < 0.001; younger group for r = 0.28, *p* < 0.001), general health (middle-aged group for *r* = 0.22, *p* < 0.01; younger group for *r* = 0.31, *p* < 0.001), and motivation to continue working (middle-aged group for *r* = 0.15, *p* < 0.05; younger group for *r* = 0.20, *p* < 0.01). Downgrading expectation was not positively related to any outcome variables. For the younger group, it can be seed that help-seeking was positively associated with general health (*r* = 0.19, *p* < 0.01), but it was not positively associated for the middle-aged group (*r* = 0.03, *p* = 0.67). It was a crucial difference between the two groups. Another difference is gender; for the middle-age group, gender was negatively related with help-seeking (*r* = −0.14, *p* < 0.05), but for the younger group, gender was positively related to downward social comparison (*r* = 0.16, *p* < 0.05).

### 3.2. Confirmatory Factor Analyses

Prior to the incremental validity testing, confirmatory factor analysis was performed to provide evidence that the focal predictor and covariate were separate constructs to examine the construct validity [70]. In the CFA, we included the eight study variables and their indicators: job crafting (three parcel items), help-seeking (three items), positive reappraisal (three items), downward social comparison (four items), downgrading expectation (three items), work engagement (three items), general health (five items), and motivation to continue working after retirement (three items). For the confirmatory factor analysis model comparisons, see Table 4.

This study assessed goodness of fit with fit indices, including χ^2^*/df*, CFI, TLI, RMSEA, and SRMR, using recommendations with a Chi-Squared/*df* < 3 [71], CFI value above 0.90 [71], TLI value close to 0.90 or 0.95 [71], RMSEA < 0.07 [72], and a cutoff value close to 0.08 for SRMR [73]. This eight-factor model showed an adequate fit to the data (χ^2^*/df* = 2.69, CFI = 0.925, TLI = 0.911, RMSEA = 0.066, SRMR = 0.050).

### 3.3. Hypothesis Testing

Given the focus on incremental validity, a series of hierarchical regression analyses and SEM were used. To avoid concerns regarding the multicollinearity issue, predictor variables were converted into standardised z-scores. Each hierarchical regression analysis included three steps. In step 1, the control variable (gender) was entered for the first block (Model 1; M1). In step 2, job crafting was entered for the second block (Model 2; M2). In the following step, each control strategy was entered for the third block (Model 3; M3). In step 3, SEM was used to offer alternative evidence that could remedy the potential problems of multiple regression [70]. Each SEM analysis included the control variable (gender), job crafting, and each strategy to predict each outcome variable.

Hypothesis 1 stated that job crafting is positively related to each dimension of healthy ageing at work for middle-aged education professionals. As can be seen from Table 5, the results of the hierarchical regression analysis support H1a (*b* = 0.35, *p* < 0.001) for work engagement, H1b (*b* = 0.14, *p* < 0.01) for general health, and H1c (*b* = 0.28, *p* < 0.001) for motivation to continue working after retirement. The results also indicate that job crafting is positively related to each dimension of healthy ageing at work for younger education professionals.

Hypothesis 2 stated that help-seeking has incremental validity predicting each dimension of healthy ageing at work beyond job crafting for middle-aged education professionals. As shown in Table 5, there was no significant incremental effect of help-seeking among middle-aged professionals predicting work engagement (∆*R*^2^ = 0.01, *F*_change_ (1, 187) = 2.10, *p* = 0.15), general health (∆*R*^2^ = 0.00, *F*_change_ (1, 187) = 0.43, *p* = 0.51), and motivation to continue working after retirement (∆*R*^2^ = 0.00, *F*_change_ (1, 187) = 0.60, *p* = 0.44). Hypotheses 2a–c were not supported. Similarly, help-seeking has incremental validity predicting each dimension of healthy ageing at work beyond job crafting for younger professionals.

In support of Hypotheses 3a, 3b, and 3c, positive reappraisal had incremental validity predicting work engagement (∆*R*^2^ = 0.09, *F*_change_ (1, 187) = 20.70, *p* < 0.001), general health (∆*R*^2^ = 0.16, *F*_change_ (1, 187) = 36.07, *p* < 0.001), and motivation to continue working after retirement (∆*R*^2^ = 0.03, *F*_change_ (1, 187) = 5.72, *p* < 0.05) for middle-aged education professionals (See Table 6). On the contrary, positive reappraisal had no incremental validity predicting each dimension of healthy ageing at work beyond job crafting for younger professionals.

Hypotheses 4a and 4b stated that downward social comparison has incremental validity predicting work engagement and general health beyond job crafting for middle-aged education professionals, respectively. Our results lend support for Hypothesis 4a (∆*R*^2^ = 0.02, *F*_change_ (1, 187) = 5.12, *p* < 0.05; see Table 7) and Hypothesis 4b (∆R^2^ = 0.02, *F*_change_ (1, 187) = 4.91, *p* < 0.05; see Table 7). In contrast, downward social comparison had no incremental validity predicting each dimension of healthy ageing at work beyond job crafting for younger professionals.

Hypothesis 4c stated that downgrading expectation has incremental validity predicting general health beyond job crafting for middle-aged teachers. Thus, Hypothesis 4c was not supported (∆R^2^ = 0.00, *F*_change_ (1, 187) = 0.00, *p* = 0.983; see Table 8). Likewise, downgrading expectation had no incremental validity predicting general health beyond job crafting for younger group.

### 3.4. Supplemental Analysis

Given the recommendations from Wang and Eastwick [70], we used SEM to test the complete model instead of part of the model using the multiple regression approach for the middle-aged group and younger group.

**Figure 1:**
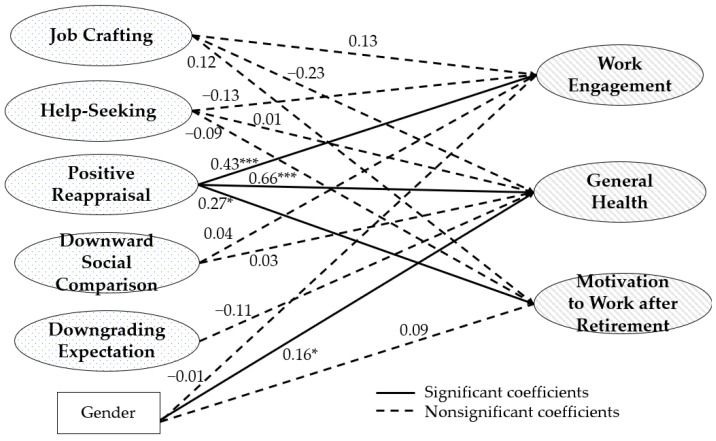
SEM path analysis results for middle-aged group. * *p* < 0.05; *** *p* < 0.001.

The model for middle-aged group with adequate fit (χ^2^/*df* = 1.95, CFI = 0.905, TLI = 0.889, RMSEA = 0.070, SRMR = 0.060). The latent variables representing positive reappraisal significantly predicted work engagement (β = 0.43, *SE* = 0.12, *p* < 0.001; see Figure 1), general health (β = 0.66, *SE* = 0.14, *p* < 0.001), and motivation to continue working after retirement (β = 0.27, *SE* = 0.12, *p* < 0.05), whereas other latent variables did not predict these significantly. The determinant of healthy ageing at work among middle-aged education professionals is positive reappraisal as a selective secondary control. In addition, gender significantly predicted general health (β = 0.16, *SE* = 0.07, *p* < 0.05).

**Figure 2:**
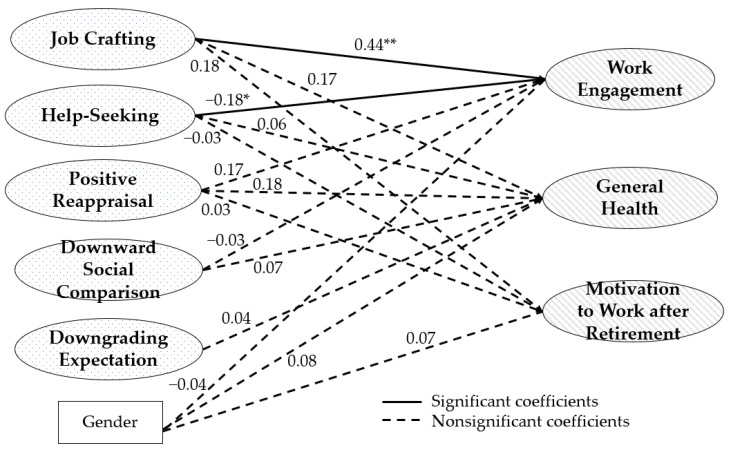
SEM path analysis results for younger group. * *p* < 0.05; ** *p* < 0.01.

Moreover, we also provided the results of the model for the younger group with adequate fit (χ^2^/*df* = 1.99, CFI = 0.910, TLI = 0.895, RMSEA = 0.071, SRMR = 0.065). The latent variables representing job crafting significantly predicted work engagement (β = 0.44, *SE* = 0.16, *p* < 0.01; see Figure 2). In addition, the latent variables representing help-seeking significantly predicted work engagement (β = −0.18, *SE* = 0.09, *p* < 0.05) with a negative coefficient, whereas other latent variables did not predict any outcome variables significantly. The determinant of healthy ageing at work among younger education professionals is job crafting as a selective primary control.

## 4. Discussion

The aim of this study was to identify the influence of job crafting and other control strategies on healthy ageing at work. The present study advances occupational health research using a life span development perspective by examining the incremental validity of different control strategies predicting the healthy ageing of education personnel at work beyond job crafting. Moreover, we also tested SEM to provide more evidence of the determinants of healthy ageing at work for different age groups—middle-aged and younger—of education professionals.

Job crafting as a selective primary control was positively related with each dimension of healthy ageing at work among middle-aged and younger education professionals. These findings are in line with previous research [11,34,38]. On the other hand, the determinant of healthy ageing at work among middle-aged education professionals is positive reappraisal, while the determinant of healthy ageing at work among younger education professionals is job crafting, according to the results of the SEM. This study used utilisation crafting and development crafting as the representatives of job crafting. The characteristics of education professionals require interaction with people (students, colleagues, parents), which can be explored more in the future in relation to relational crafting and healthy ageing at work. For example, older workers may become more selective about who they invest time in interacting with at work and may distance themselves from colleagues who do not share the same core values [10].

Our results show that positive reappraisal had incremental validity for each dimension of healthy ageing at work, and it is also a key strategy for the middle-aged group. Similarly, this is consistent with previous research on teacher well-being [41,42,43] and health status [43]. Positive reappraisal is an established coping strategy for the ageing process for the older population [52]. Moreover, the incremental validity of downward social comparison was also supported from our findings and is consistent with previous studies [42,48]. Based on the perspectives from the MTD to explain why positive reappraisal and downward social comparison as secondary control strategies play key roles among middle-aged teachers, secondary control increased in midlife and reached its highest level in older age to enhance primary control [5,32]. It is worth noting, however, that according to the results of the SEM, positive reappraisal was more critical for the middle-aged group than downward social comparison; positive reappraisal as a selective secondary control operated primarily as a mechanism for goal engagement from the MTD [30], perhaps explaining why positive reappraisal plays a key role in healthy ageing at work.

This study revealed that help-seeking as a compensatory primary control strategy had no incremental validity for healthy ageing at work for the middle-aged group. Some possible reasons for this difference in the findings might be that help-seeking might diminish autonomy [74] and might be difficult to do with different occupations [32]. Data show that help-seeking behaviours are less frequent among older teachers [53]. We also found no support for the incremental validity of downgrading expectation as a compensatory secondary control strategy. Cultural differences in regard to reliance on secondary control strategies could help to explain this [5]; Asian teachers may not use secondary control, compared to other cultural groups, because of a different relationship with self-esteem. Another possible reason—specifically for the older adults reporting better health—is that they have little need for the use of downgrading expectation and would use primary control strategies instead [75].

The results for the younger group show that other strategies (help-seeking, positive reappraisal, downward social comparison, and downgrading expectation) did not have incremental validity beyond job crafting; therefore, job crafting was the key strategy in predicting their healthy ageing at work, especially for work engagement.

As with all studies, our results must be interpreted with caution due to the limitations of the methods we used. Firstly, our work is a cross-sectional study, analysing data from a population at a single point in time. Since healthy ageing at work is a long-term process, longitudinal studies are needed to better understand the control strategies used by middle-aged education personnel. As several cohorts of teachers are currently being followed around the world, we might suggest that some items dedicated to end-of-career control strategies could be included in the data being collected [76]. Secondly, we did not include contextual variables concerning schools, and we recognise that it is likely that local factors are involved in the way in which professionals use control strategies. Thirdly, despite CFA and SEM being used to solve the potential problems of multiple regression analysis in this study, future research could test the incremental validity on models designed to test multiple predictors [77]. Fourthly, we defined middle-aged as 45–65-years-old in our study in response to the policy in Taiwan, and future research could explore future ageing labour trends in other age ranges (e.g., over 65-years-old). Although we treated gender as a control variable, gender was negatively related with help-seeking for the middle-age group, but gender was positively related to downward social comparison for the younger group (Gender is coded 0 = female, 1= male). In another study of Chipperfield [78], women use compensatory secondary control strategies more frequently than men. Further studies are needed to better understand the influence of gender on coping practices. Finally, the analysis showed job crafting was significant in Model 2 with predicted general health and became insignificant in Model 3 with the additional variables of positive reappraisal and downward social comparison. In addition to representing incremental validity, the possibility of a mediating effect is worth investigating in future studies to gain a more comprehensive understanding of the MTD perspective.

It is possible to identify some practical implications of our work. First of all, evidence-based interventions aiming to help people to cope with challenges, promoting healthy ageing at work, must be actively developed within the system [5]. These could include designing behaviour change, encouraging goal adjustment, and helping to identify more control strategies [25]. Secondly, the career development of older workers should not be neglected, to specifically promote healthy ageing at work and a good work–retirement transition [79].

This study provides some resources and information that could be used by career development practitioners within counselling. For example, a career development advisor/counsellor could invite their client to answer the questionnaire to know their degree of use of each strategy compared with the norm. Then they could provide tailored options for future coping strategies on healthy ageing at work according to this study’s results, such as the functions of the secondary control strategy for middle-aged education professionals.

Finally, meaningful integration of career development and human resource development should be considered within education [80]. A career-crafting training program is required to encourage education personnel to develop their lifelong career properly and consciously. For example, van Leeuwen et al. [81] designed career crafting training, which included a mix of theory, reflection, and exercises. Their intervention enhanced career self-management perceptions and job crafting behaviour. Further work to integrate this study and career crafting training could introduce the motivational theory of life span development and each control strategy and optimisation (theory), encourage experience sharing (reflection), and enable the design of a future action plan (exercises).

## 5. Conclusions

The ageing of societies is a global phenomenon, even if it is more or less marked in different countries. It is leading to upheavals in all aspects of our society, and particularly at work. The lengthening of the life span leads to the need to maintain the motivation, competence, and productivity of professionals over a longer period. Supporting them in the midlife period calls for the development of appropriate human resource structures and interventions. Generating data on occupational health in this life span period is likely to lead to renewed approaches to adapt to the needs of these workers. The main contribution of this study to the literature is the provision of the evidence based on the motivational theory of life span development that, on the one hand, job crafting as a selective primary control strategy is associated with all dimensions of healthy ageing at work, and, on the other hand, that positive reappraisal as a selective secondary control strategy is a critical influence among the middle-age group and, for the younger group, job crafting plays an important role. These results contribute to a better understanding of how education staff mobilise control strategies and offer insights into the development of support systems during this important period in their careers.

## Figures and Tables

**Table 2:** Resources for measurement, sample items, and reliability of variables.

Variables Category	Measure	Sample Items and Scales	Cronbach’s α
Predictor Variables	Job crafting [58]	“I change my job to use my current knowledge and capacities to the fullest” (utilisation crafting), and “I take on more responsibilities” (developmental crafting) (1 = never, 5 = always)	0.92
	Help-seeking [39]	“Find someone else to help with the task” (1 = never, 5 = always)	0.83
	Positive reappraisal [39]	“Look for what you can learn from the difficulty” (1 = never, 5 = always)	0.87
	Downward social comparison [39]	“Tell yourself that others have worse problems” (1 = never, 5 = always)	0.88
	Downgrading expectation [39]	“Tell yourself that you mustn’t set your goals too high” (1 = never, 5 = always)	0.77
Outcome Variables	Work Engagement [59]	“At my work, I feel bursting with energy (Vigour)”, “I am enthusiastic about my job (Dedication)”, and “I am immersed in my work (Absorption)”. (1 = never, 7 = always).	0.90
	General Health [60]	Determined from the combination of a single-item rating (“In general, would you say your health is”; 1 = poor, 5 = excellent) and four further items (e.g., “I seem to get sick a little easier than other people”; 1 = definitely false, 5 = definitely true).	0.82
	Motivation to Continue Working after Retirement [61]	“If I were completely free to choose, I would prefer to continue working as a teacher/staff member after my retirement.” (1 = Not at all, 5 = Totally)	0.91

**Table 3:** Mean, standard deviations, and correlations among study variables.

	Variable	1	2	3	4	5	6	7	8	9	10
1.	Job Crafting	—									
2.	Help-Seeking	0.45 ***	—								
3.	Positive Reappraisal	0.70 ***	0.31 ***	—							
4.	Downward Social Comparison	0.49 ***	0.29 ***	0.59 ***	—						
5.	Downgrading Expectation	0.34 ***	0.29 ***	0.39 ***	0.41 ***	—					
6.	Work Engagement	0.39 ***	0.07	0.41 ***	0.27 ***	0.03	—				
7.	General Health	0.26 ***	0.11 *	0.37 ***	0.26 ***	0.09	0.38 ***	—			
8.	Motivation to Continue Working	0.21 ***	0.06	0.21 ***	0.17 **	0.07	0.49 ***	0.28 ***	—		
9.	Age Group	−0.01	−0.05	−0.01	0.01	0.01	0.17 **	0.05	0.15 **	—	
10.	Gender	−0.01	−0.05	−0.05	0.10 *	−0.09	−0.04	0.08	0.07	−0.01	—
*M*		3.24	2.74	3.63	3.16	3.13	4.73	3.27	3.28	0.49	0.33
*SD*		0.82	0.91	0.89	0.91	0.84	0.99	0.73	1.04	0.50	0.47

*Note. N* = 386. * *p* < 0.05; ** *p* < 0.01; *** *p* < 0.001. Age group is coded 0 = younger group (22–44-years-old), 1 = middle-aged group (45–65-years-old). Gender is coded 0 = female, 1 = male.

**Table 4:** Confirmatory factor analysis model comparisons.

Model	Factor Structure	χ^2^	*df*	χ^2^*/df*	CFI	TLI	RMSEA	SRMR
8-factor	1, 2, 3, 4, 5, 6, 7, 8	796.25	296	2.69	0.925	0.911	0.066	0.050
7-factor Model 1	1 + 2, 3, 4, 5, 6, 7, 8	1134.39	303	3.74	0.875	0.856	0.084	0.064
7-factor Model 2	1 + 3, 2, 4, 5, 6, 7, 8	1051.65	303	3.47	0.888	0.870	0.080	0.059
7-factor Model 3	1 + 4, 2, 3, 5, 6, 7, 8	1346.18	303	4.44	0.844	0.819	0.094	0.072
7-factor Model 4	1 + 5, 2, 3, 4, 6, 7, 8	1079.29	303	3.56	0.884	0.865	0.081	0.081
7-factor Model 5	2 + 3, 1, 4, 5, 6, 7, 8	1207.40	303	3.98	0.864	0.843	0.088	0.068
7-factor Model 6	2 + 4, 1, 3, 5, 6, 7, 8	1246.39	303	4.11	0.859	0.836	0.090	0.074
7-factor Model 7	2 + 5, 1, 3, 4, 6, 7, 8	1091.97	303	3.60	0.882	0.863	0.082	0.073
7-factor Model 8	3 + 4, 1, 2, 5, 6, 7, 8	1128.33	303	3.72	0.876	0.857	0.084	0.059
7-factor Model 9	3 + 5, 1, 2, 4, 6, 7, 8	1038.52	303	3.43	0.890	0.872	0.079	0.063
7-factor Model 10	4 + 5, 1, 2, 3, 6, 7, 8	1022.72	303	3.38	0.892	0.875	0.078	0.061
7-factor Model 11	6 + 7, 1, 2, 3, 4, 5, 8	1310.56	303	4.33	0.849	0.825	0.093	0.078
7-factor Model 12	6 + 8, 1, 2, 3, 4, 5, 7	1411.719	303	4.66	0.834	0.807	0.097	0.066
7-factor Model 13	7 + 8, 1, 2, 3, 4, 5, 6	1579.325	303	5.21	0.809	0.778	0.104	0.078

*Note.* Factor 1 = Job Crafting. Factor 2 = Help-Seeking. Factor 3 = Positive Reappraisal. Factor 4 = Downward Social Comparison. Factor 5 = Downgrading Expectation. Factor 6 = Work Engagement. Factor 7 = General Health. Factor 8 = Motivation to continue working after retirement. CFI = comparative fit index. TLI = Tucker–Lewis index. RMSEA = root-mean-square error of approximation. SRMR = standard root-mean-square residual.

**Table 5:** Results from hierarchical regression analyses of Help-Seeking predicting healthy ageing at work.

	Work Engagement			General Health			Motivation to Continue Working after Retirement		
Predictor	M1	M2	M3	M1	M2	M3	M1	M2	M3
Middle-aged group (*N* = 191)									
Gender	−0.09	−0.03	−0.06	0.13	0.15	0.14	0.15	0.19	0.18
Job Crafting		0.35 ***	0.40 ***		0.14 **	0.16 **		0.28 ***	0.31 ***
Help-Seeking			−0.11			−0.04			−0.07
∆*R*^2^	0.00	0.13 ***	0.01	0.01	0.04 **	0.00	0.00	0.07 ***	0.00
∆*F*	0.38	27.88 ***	2.10	1.22	6.88 **	0.43	0.78	14.18 ***	0.60
Younger group (*N* = 195)									
Gender	−0.07	−0.12	−0.11	0.14	0.12	0.11	0.16	0.14	0.14
Job Crafting		0.43 ***	0.48 ***		0.24 ***	0.23 ***		0.16 *	0.16 *
Help-Seeking			−0.13			0.03			0.00
∆*R*^2^	0.00	0.18 ***	0.01	0.01	0.12 ***	0.00	0.01	0.03 *	0.00
∆*F*	0.19	42.96 ***	2.99	1.77	25.67 ***	0.40	1.07	5.34 *	0.00

*Note.* * *p* < 0.05; ** *p* < 0.01; *** *p* < 0.001. The regression coefficients were unstandardised. The middle-aged group = 45–65-years-old, younger group = 22–44-years-old. Gender is coded 0 = female, 1 = male.

**Table 6:** Results from hierarchical regression analyses of Positive Reappraisal predicting healthy ageing at work.

	Work Engagement			General Health			Motivation to Continue Working after Retirement		
Predictor	M1	M2	M3	M1	M2	M3	M1	M2	M3
Middle-aged group (*N* = 191)									
Gender	−0.09	−0.03	0.00	0.13	0.15	0.19	0.15	0.19	0.21
Job Crafting		0.35 ***	0.11		0.14 **	−0.10		0.28 ***	0.14
Positive Reappraisal			0.40 ***			0.42 ***			0.25 *
∆*R*^2^	0.00	0.13 ***	0.09 ***	0.01	0.04 **	0.16 ***	0.00	0.07 ***	0.03 *
∆*F*	0.38	27.88 ***	20.70 ***	1.22	6.88 **	36.07 ***	0.78	14.18 ***	5.72 *
Younger group (*N* = 195)									
Gender	−0.07	−0.12	−0.10	0.14	0.12	0.13	0.16	0.14	0.14
Job Crafting		0.43 ***	0.30 **		0.24 ***	0.15 ***		0.16 *	0.13
Positive Reappraisal			0.16			0.12			0.04
∆*R*^2^	0.00	0.18 ***	0.01	0.01	0.12 ***	0.01	0.01	0.03 *	0.00
∆*F*	0.19	42.96 ***	2.97	1.77	25.67 ***	3.13	1.07	5.34 *	0.18

*Note.* * *p* < 0.05; ** *p* < 0.01; *** *p* < 0.001. The regression coefficients were unstandardised. The middle-aged group = 45–65-years-old, younger group = 22–44-years-old. Gender is coded 0 = female, 1 = male.

**Table 7:** Results from hierarchical regression analyses of Downward Social Comparison predicting work engagement and general health.

	Work Engagement			General Health		
Predictor	M1	M2	M3	M1	M2	M3
Middle-aged group (*N* = 191)						
Gender	−0.09	−0.03	−0.06	0.13	0.15	0.13
Job Crafting		0.35 ***	0.29 ***		0.14 **	0.09
Downward Social Comparison			0.16 *			0.13 *
∆*R*^2^	0.00	0.13 ***	0.02 *	0.01	0.04 **	0.02 *
∆*F*	0.38	27.88 ***	5.12 *	1.22	6.8 8 **	4.91 *
Younger group (*N* = 195)						
Gender	−0.07	−0.12	−0.13	0.14	0.12	0.09
Job Crafting		0.43 ***	0.40 ***		0.24 ***	0.18 **
Downward Social Comparison			0.05			0.11
∆*R*^2^	0.00	0.18 ***	0.00	0.01	0.12 ***	0.01
∆*F*	0.19	42.96 ***	0.28	1.77	25.67 ***	2.89

*Note.* * *p* < 0.05; ** *p* < 0.01; *** *p* < 0.001. The regression coefficients were unstandardised. The middle-aged group = 45–65-years-old, younger group = 22–44-years-old. Gender is coded 0 = female, 1 = male.

**Table 8:** Results from hierarchical regression analyses of Downgrading Expectation predicting general health.

	General Health		
Predictor	M1	M2	M3
Middle-aged group (*N* = 191)			
Gender	0.13	0.15	0.15
Job Crafting		0.14 **	0.14 *
Downgrading Expectation			−0.00
∆*R*^2^	0.01	0.04 **	0.00
∆*F*	1.22	6.88 **	0.00
Younger group (*N* = 195)			
Gender	0.14	0.12	0.12
Job Crafting		0.24 ***	0.24 ***
Downgrading Expectation			0.01
∆*R*^2^	0.01	0.12 ***	0.00
∆*F*	1.77	25.67 ***	0.00

*Note.* * *p* < 0.05; ** *p* < 0.01; *** *p* < 0.001. The regression coefficients were unstandardised. The middle-aged group = 45–65-years-old, younger group = 22–44-years-old. Gender is coded 0 = female, 1 = male.

## Data Availability

The data are not publicly available due to the conditions specified in the ethics application.
